# Professional Athletes Maintain High TNF-Alpha or IFN-Gamma Related Inflammatory Status after Recovering from COVID-19 Infection without Developing a Neutralizing Antibody Response

**DOI:** 10.3390/sports11050097

**Published:** 2023-04-30

**Authors:** Mira Ambrus, Eszter Fodor, Timea Berki, Veronika Müller, Ádám Uhlár, István Hornyák, Zsombor Lacza

**Affiliations:** 1 Research Center for Sport Physiology, Hungarian University of Sports Sciences, 1123 Budapest, Hungary; 2 Department of Immunology and Biotechnology, Faculty of Medicine, University of Pécs, 7624 Pécs, Hungary; 3 Clinic of Pulmonology, Semmelweis University, 1083 Budapest, Hungary; 4 Institute of Translational Medicine, Semmelweis University, 1094 Budapest, Hungary

**Keywords:** SARS-CoV-2, COVID-19, sports, Hungarian national teams

## Abstract

Introduction: Professional athletes are endangered by COVID-19 and belong to the high-risk population due to their lifestyle. To obtain information on the behavior of COVID-19 in professional athletes, serological, cytokine, and virus neutralization capacities were analyzed. Materials and methods: Hungarian national teams participated in international sports events during the early phases of the COVID-19 epidemic in 2020. Altogether, 29 professional athletes volunteered to donate plasma. Their serological status was evaluated by IgA, IgM, and IgG ELISAs and the highest virus neutralization titer in an in vitro live tissue assay. Plasma cytokine patterns were analyzed with a Bioplex multiplex ELISA system. Results: Surprisingly, only one athlete (3%) had anti-SARS-CoV-2 IgG, while IgA was more common (31%). Neither plasma showed direct virus neutralization in a titer over 1:10; hence, they were not suitable for reconvalescent treatment. The ‘cytokine storm’ markers IL-6 and IL-8 were at baseline levels. In contrast, either the TNF-alpha-related cytokines or the IFN-gamma-associated cytokines were elevated. There was a strong negative correlation between the TNF-alpha- or IFN-gamma-related cytokines. Conclusions: Professional athletes are susceptible to the SARS-CoV-2 infection without developing long-term immunity through neutralizing immunoglobulins. Elevated secretory and cellular immunity markers indicate that these systems are probably responsible for virus elimination in this subpopulation.

## 1. Introduction

Professional athletes, such as National Olympic Teams, form a special cohort that is highly affected by outbreaks of epidemics such as COVID-19 [1]. Athletes live a mobile lifestyle that makes them a high-risk population for both getting infected by the virus and also for transmitting it to their peers [2]. They often travel from all over the world to training camps and competitions that are held in mild climates, i.e., Italy or Spain—the two original focal points of the SARS-CoV-2 pandemic in Europe [3]. In addition to co-location with hotspots, athletes have an increased risk of COVID-19 transmission during sports activities. They train in teams and engage in physical contact even in individual sports. There is a tendency to not adhere to guidelines for social distancing or personal hygiene, and they use shared equipment and common sanitary facilities, which further increase the risk of disease transmission. However, there is very limited data published regarding the prevalence, nature, and behavior of COVID-19-related illness in the professional athlete population [2]. Policy decisions are best made when reliable data is readily available and allows for mathematical predictions or at least identifiable trends. However, the speed of the COVID-19 pandemic did not allow for gathering data from the general population, let alone specific cohorts such as professional athletes. Important decisions, such as postponing the 2020 Tokyo Olympics, were made under public pressure rather than exact epidemiological calculations, and their effectiveness is being evaluated ex-post [4,5,6]. There is a very strong incentive in the sports industry to keep up the level and mode of sports activities without disruptions since the financing of the value chain is heavily dependent on actual participation at games. Health risks may be secondary in the minds of sports professionals in the same manner as it is the case with, e.g., performance enhancers, so it is expected that despite medical concerns for a safe return to play, professional sports restart earlier than what is allowed for the general public [7]. Indeed, it was allowed at the 2022 Tour de France to continue racing even after a cyclist tested positive for COVID-19. Therefore, it is important to understand indepth the effect of SARS-CoV-2 infection on professional athletes to aid individual back-to-sport and policy-level decisions.

There is evidence that young individuals with SARS-CoV-2 infection develop relatively mild symptoms and recover almost completely over 5–7 days [8]. However, a minority of those infected have a heightened risk of further deterioration between days 7 and 9, with more fulminant lower respiratory tract manifestations and a possible systemic infection [8]. In susceptible patients, an uncontrolled inflammatory immune response leads to extremely high levels of pro-inflammatory cytokines such as IL-6, IL-8, or TNF alpha [9]. This explosion of selective cytokines is termed ‘cytokine storm’, which triggers or at least signals the consequential acute respiratory distress syndrome (ARDS) and multiple organ failure [10]. These are, in turn, responsible for the lethality of SARS-CoV-2 infection, such as in earlier coronavirus epidemics, namely the SARS-CoV-2 in 2002 and the MERS-CoV in 2012 [11]. Therefore, measuring the levels of inflammatory cytokines has become a way of monitoring infected individuals, but, without clear correspondence with symptoms and progression [12]. On the other hand, elite athletes have an altered inflammatory state mainly due to myokine production during strenuous training—interestingly, the main elevated myokines are IL-6, IL-8, and TNF-alpha, the very same molecules that are elevated in acute COVID-19 disease [13,14]. Therefore, it may be hypothesized that professional athletes who are in a highly trained state may respond differently than the average population.

Next to the approved antivirals or vaccines against SARS-CoV-2 infection, the healthcare systems resort to the transfusion of hyper-immune or convalescent plasma derived from recovered donors to treat severely ill patients [15,16,17,18,19,20,21]. Convalescent plasma (CP) can act through virus-specific neutralizing antibodies, which are mainly IgG and IgM isotypes, although IgA is also important in mucosal viral defense. Moreover, the anti-inflammatory effect of CP can also control the overactivated immune system of severely ill patients [22]. Both the Food and Drug Administration (FDA) and the European Medicinal Agency (EMA) have issued guidelines and set up expedited approval protocols for CP therapies as the first available antiviral therapy (Investigational COVID-19 Convalescent Plasma; Guidance for Industry). A campaign for plasma donations was met with a strong response from the Hungarian national teams, which had athletes diagnosed with SARS-CoV-2 infection in the early weeks of the pandemic and who were already fully recovered and eager to help. This allowed the collection of data from a well-defined sub-population from five Olympic disciplines: swimming, kayaking, wrestling, fencing, and biking. We investigated the SARS-CoV-2 infection-induced immune and inflammatory responses of these top athletes, who were fully prepared to take part in the 2020 Tokyo Olympics, to understand how this very special subpopulation responds to the pandemic.

## 2. Materials and Methods

### 2.1. Subjects and Plasma Sample Isolation

Members of the Hungarian National Teams for swimming, fencing, wrestling, mountain biking, and kayaking were offered testing for SARS-CoV-2 by their respective associations and clubs after it became evident that the COVID-19 epidemic had reached Europe. All sportsmen were exposed to viral infections in 2 March 2020 during international sports events. Swimmers were in Thailand, South Africa, Turkey, and the USA in international training camps and competitions; fencers participated at the World Championship in Budapest; wrestlers at the European Championships in Italy, bikers in the Olympic qualification race in Turkey, and the kayakers in an international training camp in Hungary. The presence of virus RNA in oral or nasal mucosal swabs was measured with PCR. Participation in the testing was voluntary, and of those 97 who underwent testing, 11 had a positive PCR result. As this happened during the first wave of the COVID-19 epidemic, some athletes with typical symptoms and previous contact with positively tested individuals were not confirmed with PCR at the time. Nonetheless, all those who had typical COVID-19 symptoms or tested positive signed up for plasma donation and were included in the study (N = 29).

Blood samples were obtained from recovered donors of both genders aged 18–34 years in 3.5 mL VACUETTE blood collection tubes with 3.2% sodium citrate (Greiner-Bio-One) under ethical approval (IRB approval number IV/3457/2-2020-EKU, ClinicalTrials.gov Identifier: NCT04345679). A plasma sample of 1.5 mL was isolated from each donor by centrifuging whole blood at 3000 rpm for 5 min. Samples were stored at 80 °C until measurements. All athletes were at the top of their game; our cohort included Olympic, World-, and European Championship medalists, and none of them retired from professional sports at the time of testing.

### 2.2. Measurement of Virus Neutralizing Titer

VeroE6 cells (an African green monkey kidney cell line) were grown in Dulbecco’s modified eagle medium DMEM (Lonza), supplemented with 2% fetal bovine serum (FBS) (EuroClone), and 1% Penicillin–Streptomycin (Lonza), and maintained at 37 °C in a humidified incubator at 5% CO_2_. SARS-CoV2 was isolated in a Biosafety Laboratory Level 4, and the viral titer of the stock was determined by a TCID50 assay (University of Pécs, National Virology Laboratory). During neutralization, 50 µL two-fold serially diluted and heat-treated (56 °C, 30 min) sera were incubated with 50 µL DMEM containing 100 TCID50 of SARS-CoV-2 for one hour at 37 °C in 96-well microtiter plates. Positive controls did not contain serum, and negative controls contained only DMEM. Confluent VeroE6 cells were infected with 100 µL two-fold serially diluted neutralized virus for 30 min. After the infection, 150 µL sustaining media was added to the cells and incubated for 3 days at 37 °C and 5% CO_2_. The sera neutralization titer was determined by calculating the highest dilution of sera that prevents infection in 50% of replicate inoculations.

### 2.3. Detection of Anti-SARS-CoV-2 Immunoglobulins, Cytokines and Virus RNA

The presence of IgG and IgM against the SARS2-CoV-2 nucleocapsid antigen (Microgen or Genetics) and the level of IgG and IgA against the spike protein antigen (Euroimmune) were determined in the plasma samples by semi-quantitative ELISA assays. SARS2-CoV-2 RNA was detected in the mucosal swab samples by standard clinical laboratory assays at registered diagnostic centers. The levels of 27 inflammatory cytokines MIP-1beta, IL-6, IFN-gamma, IL-1ra, IL-5, GM-CSF, TNF-alpha, RANTES, IL-2, IL-1beta, Eotaxin, BFGF, VEGF, PDGF-BB, IP-10, IL-13, IL-4, MCP-1, IL-8, MIP-1alpha, IL-10, G-CSF, IL-15, IL-7, IL-12, IL-17, and IL-9 were measured in the plasma samples by Bio-PlexTM Human Cytokine Assay #M500KCAF0Y (Biorad).

## 3. Statistical Analysis

The dataset was not normally distributed on the basis of the D’Agostino and Pearson omnibus normality tests. Pearson correlation values are considered ‘strong’ where the coefficient is over 0.7 and ‘very strong’ where the coefficient is over 0.9. Prism 8 software was used for statistical analysis.

## 4. Results

**Figure 1:**
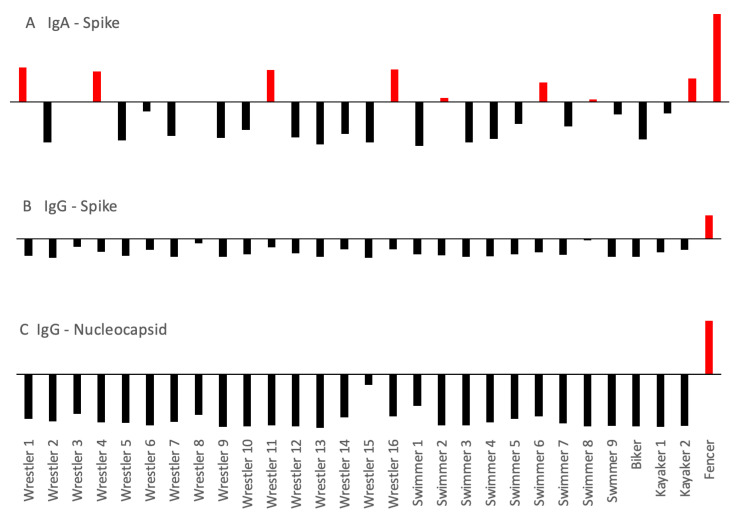
Immunoglobulin levels of professional athletes 6–8 weeks after SARS-CoV-2 infection. Positive values over the manufacturer-set threshold are shown in red, negative values are in black, and the threshold is on the *x*-axis. Panel (**A**) shows anti-SARS-CoV-2 spike protein IgA in the circulating plasma. Panels (**B**,**C**) show anti-SARS-CoV-2 spike or nucleocapsid protein IgG levels, respectively. Note that only one athlete was positive for all three types of immunoglobulins, while IgA was positive in 7 out of 29, albeit at a low level.

We measured the virus-neutralizing titer of plasma samples with a direct neutralization assay of SARS-CoV-2 proliferation in infected tissues. Surprisingly, not one of them had a strong or even moderate neutralizing titer, as all were under 1:10, defined as the highest dilution of neutralizing plasma added to SARS-CoV-2 infected tissues. As a cross-reference, we measured the specific anti-SARS-CoV-2 IgG levels with ELISA in the same set of donors and found that only one athlete had a positive IgG level, supporting the neutralization data (Figure 1). Antibodies against the spike and the nucleocapsid proteins were in concert with each other. None of the athletes were positive for IgM. In order to investigate the respiratory immune system’s response against the virus, we evaluated plasma anti-SARS-CoV-2 IgA levels. Interestingly, 31% of the cohort tested positive for IgA, indicating that the mucosal immune system was still active even 4–6 weeks after inoculation (Figure 1).

**Figure 2:**
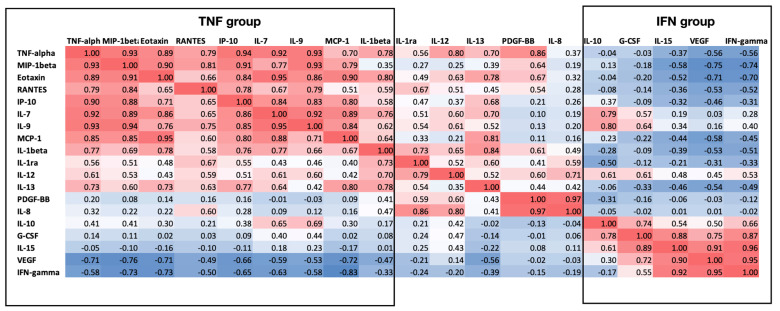
Cytokine levels of professional athletes 6-8 weeks after SARS-CoV-2 infection. A correlation heat map of all measured cytokines was detectable in at least one subject. Red color indicates positive correlation, blue color indicates negative correlation, with numbers in the squares representing Pearson correlation coefficients (r^2^). Black brackets highlight two easily recognizable groups of cytokines whose concentrations are strongly linked to each other. Note the high occurrence of very strong correlation levels, i.e., those above 0.8.

**Figure 3:**
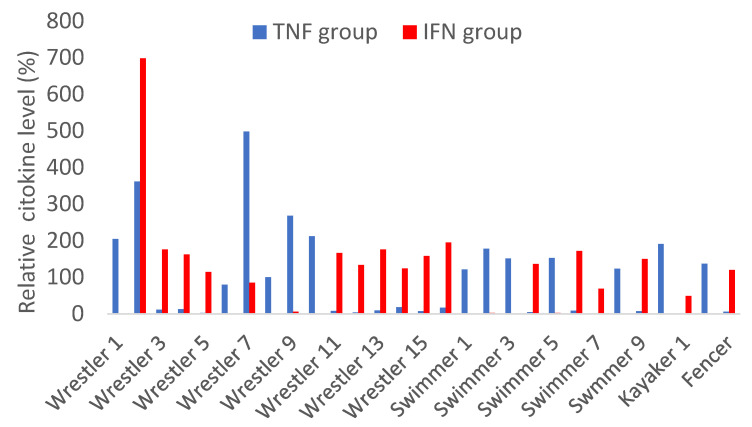
Relative cytokine levels of professional athletes 6-8 weeks after SARS-CoV-2 infection. It shows the two groups, shown as surrogate variables created as average percentages of each cytokine concentration within the group. Note that almost all athletes have cytokine levels over 100%, but only in either the TNF-related (blue) or the IFN-related (red) groups, with the exception of wrestlers 2 and 7, who had elevated cytokines in both.

The cytokines IL-2, IL-4, IL-5, IL-6, IL-17, BFGF, and MIP1-α were below the detection level in all samples, indicating that even the slightest cytokine storm was not present in this population (Figure 2). Interestingly, other inflammatory markers were positive, and a clear pattern was observable in cross-correlations, as shown in Figure 2. Two groups of cytokines were discernible that strongly correlated with each other, while IL1-RA, IL-8, IL-12, IL-13, and PDGF-BB showed no correlation to either group or each other, although these were elevated in a few athletes (Figure 2). The remaining cytokines clearly belonged to two groups, either correlating with TNF-α levels or with IFN-gamma levels. Cytokines correlating with TNF-α are MIP1-beta, Eotaxin, RANTES, IP-10, IL-7 and 9, MCP-1, and IL1-beta. The other end of the spectrum is the group consisting of IFN-gamma, VEGF, IL-10, IL-15, and G-CSM (Figure 2). What is more striking in the dataset is that these two groups are inversely correlated with each other. In other terms, if the TNF-related cytokines are elevated, the IFN-related ones are reduced. Indeed, with the exception of two athletes where both the IFN and TNF groups of cytokines were elevated (wrestlers 2 and 7), a strong inverse correlation is observed in all the other athletes (Figure 3). Although these are not diagnostic but research-level tests with no accepted standard values, it can be noticed that either one or the other group is elevated in each athlete, i.e., not one subject in our cohort has low cytokine values across the board. If we compare the immune status and the inflammatory status of the athletes, no association can be seen: those with elevated IgA or IgG levels have either high TNF or IFN-associated cytokines, without any clear distinction or correlation (Figure 1). For example, the one fencer with the outlying and strong immunoglobulin levels had an elevated IFN-related cytokine response at a comparable level to several other athletes who did not have elevated immunoglobulin concentrations.

## 5. Discussion

We observed that over 10% of professional athletes who were exposed to the first wave of the COVID-19 epidemic got infected; however, they did not develop a lasting neutralizing IgM or IgG response. In contrast to immunoglobulins, all athletes had elevated cytokine levels, with a highly distinctive pattern: either TNF-alpha-related or IFN-gamma-related cytokines were up, but not both.

Production of high-affinity anti-SARS-CoV-2 IgG by plasma cells is essential for long-term immunity and immunological memory [10,23,24]. Measurement of IgG antibodies by either quick tests providing a yes or no answer from a drop of blood or more accurately using ELISA kits from plasma samples is widely employed to probe the population for immunity against reinfection [25]. Surprisingly, we observed that among the athletes who recovered from SARS-CoV-2, only one potential donor had long-term serological IgG protection against the virus, but the vast majority did not. Therefore, it has to be the alternative biological defense mechanisms that successfully eliminated the virus before a proper IgG response would have been triggered. IgA is the main marker of the activated mucosal immune system. Our observation that about a third of the recovered athletes were positive for IgA indicates that the first line of defense against virus infection was indeed activated and protected the respiratory epithelium. Nonetheless, the investigated population exhibited insufficient protection, so they all have to be treated as vulnerable regardless of whether they are already through the first infection or not [26]. This is evidenced by the fact that 3 out of 11 people were reinfected with SARS-CoV-2 in the 10 months after their recovery from the first infection.

Investigating the cytokine profile of the athletes, a significantly lower level of inflammatory cytokines involved in the SARS-CoV-2 associated ‘cytokine storm’ was found. This result is expected, as the athletes in our study had mild symptoms and were far from being hospitalized. Looking deeper into the cytokine data than simple concentrations of a few selected molecules is necessary to gain meaningful insights. Various quantification techniques have a tendency to generate uncomparable results for cytokine levels; however, the multiplex assay used in the current study was also applied in previous studies, offering a direct comparison [27]. Analysis of large data matrices generated by 27 analytes in a high number of patients inevitably leads to omissions and combinations, making paper-to-paper comparison difficult—that is why we share the raw measurement data in Supplementary Table S1 to aid further research. Comparing the present data with others already available in the literature, it is apparent that the high correlation coefficients we present are way stronger than what we observed in other diseases or what is generally published with cytokine panels [28]. Specifically, this allows the recognition of meaningful cytokine-cytokine interactions that are otherwise masked in the wider variations of the non-athlete, general plasma donor population. In a study by Petrone et al., IFN-gamma levels were found to be weakly correlated to positive serology in convalescent plasma donors [29]. They also observed elevated IL-13, IFN-gamma, and MCP-1 levels in convalescent donors compared to healthy donors, which supports our current observations. Horspool et al. investigated the cytokine levels of COVID-19 patients in the acute phase of the disease with the same multiplex assay as used in the current study [27]. Interleukins 6 and 8 were elevated, as expected, as was IFN-gamma in the patients, while there was no increase in TNF-alpha or RANTES, i.e., their data also supports the negative correlation observed in the present study among the IFN- and the TNF-related cytokines. Bonny et al. also used the same assay on convalescent plasma donors and presented a complete cross-correlation heat map that is directly comparable to our data set [30]. Although similar trends may be picked up from their data than what is presented here, the strength of the correlations is way below what we observed, probably due to the demographics of the general donors vs. top athletes. The study of Petruccioli et al.—again using the same 27-cytokine panel—identified that IP-10 is associated with SARS-CoV-2 infection in both acute and convalescent patients, indicating that a cellular immune response lingers in these athletes despite their lack of seropositivity towards the virus [31]. Taken together, five cross-sectional studies using the same 27-plex cytokine assay on post-COVID patients identified similar trends; however, further research is required to uncover the time- and disease severity-dependence of each pattern—only such an extended study can lead to diagnostic-level information. Taratino et al. stated that cytokine storms may cause elevated c-reactive protein (CRP) concentrations and subsequent mitochondrial damage, which is correlated with the onset of sarcopenia in COVID-19 patients [32]. Many symptoms, especially musculoskeletal ones, persisted even for long periods of time after infection. Six months after the COVID-19 infection, patients suffered from fatigue or muscle weakness, sleep difficulties, and anxiety or depression [33].

One interpretation of the current data can be that top athletes who are clearly contaminated with the SARS-CoV-2 virus are less prone to developing a COVID-19 disease, let alone a serious one. This cannot be clearly derived from the current dataset as the study design does not allow such far-fetched conclusions; however, the results clearly point in this direction. Looking at this question from the opposite angle, i.e., that obese and sedentary people had far higher complication rates than the overall population [14], one may hypothesize that an active lifestyle and regular exercise may have some preventive effect against serious COVID-19. Interestingly, some of the main exerkines, i.e., cytokines that are released during and after exercise, such as IL-6, IL-8, IL-1ra, or TNF-alpha, are the very same ones that are elevated in COVID-19, raising the possibility that common molecular mechanisms are activated [14]. Moreover, the current study identified two separate groups of cytokines, and the one we termed the ‘TNF group’ in the current study contains several factors that are also included among exerkines in the literature, while the others in the IFN group are mainly inflammatory markers. Since these two groups of cytokines are mutually exclusive in the current dataset, it raises the possibility that the post-training state, evidenced by high exerkine levels, is a protective factor against COVID-19, which may be the aim of further studies.

The performance of the athletes was not monitored during the study as they participated in different disciplines. However, their overall performance level may not have been strongly affected by the infection, as one athlete from our cohort won a gold medal, another won a silver medal, and five others had notable final results in their respective sports. On the other hand, one serious adverse event happened after the completion of the study. Wrestlers went back to their training routines, and one athlete had a fatal cardiac arrest during a warm-up run about 10 weeks after recovery from the SARS-CoV-2 infection. It is unclear whether this sudden cardiac death is a direct consequence of the preceding viral infection; however, a higher incidence of cardiac arrest was observed in post-COVID patients [34]. In addition, long after the SARS-Cov2 outbreak, it was found that many symptoms, especially musculoskeletal ones, persist even for very long periods of time after infection, a phenomenon called ‘long COVID’ [33]. These observations highlight that COVID-19 disease among athletes, even though they may not experience severe symptoms, cannot be taken lightly.

The nature of this study has its limitations. For one, all sports investigated are individual Olympic sports, and it can be expected that team sports such as soccer or extreme sports such as rock climbing have somewhat different features related to their innate characteristics. Another bias stems from the voluntariness of plasma donations, i.e., one can investigate only those who actively signup for plasma donations after recovering from the disease. The sample size is thus arbitrary, as the study was performed on all available subjects who met the selection criteria, and as the pandemic progressed, there were many changes in testing and management of both athletes and COVID-19 patients, so there was no realistic way of increasing the sample size or repeating some experiments. Nonetheless, the present group of 29 affected individuals forms a very tight cohort of professional athletes that are otherwise hard to recruit for studies, and indeed, the current dataset revealed an unparalleled strength of correlations in this population that can be the basis for further studies. This is the first such study that provides numerical data on this population, which can be used—with wide margins—to evaluate the health risks of athletes exposed to the SARS-CoV-2 threat.

## 6. Conclusions

We observed that top athletes exposed to COVID-19 get infected easily, while at the same time, they do not develop lasting humoral immunity against reinfection. An investigation of their immunological response to the virus revealed that a strong IgA response is probably responsible for eliminating the virus at the mucosal level and thus preventing systemic antibody production. The cytokine patterns of the athletes revealed that athletes have an elevated immune status even 4–6 weeks after the infection and that it is either a general TNF-alpha-related response or a cellular immune response associated with IFN-gamma. This highlights that even asymptomatic post-COVID athletes are in an altered immunological state, which may increase their risk for other pathologies.

## Data Availability

Data is contained within the article or supplementary material. The data presented in this study are available in Table S1: Raw output data for the cytokine assay.

## Supplementary Materials

The following supporting information can be downloaded at: https://www.mdpi.com/article/10.3390/sports11050097/s1, Table S1: Raw output data for the cytokine assay.

## References

[1] Agha-Alinejad H., Ahmadi Hekmatikar A.H., Ruhee R.T., Shamsi M.M., Rahmati M., Khoramipour K., Suzuki K. (2022). A Guide to Different Intensities of Exercise, Vaccination, and Sports Nutrition in the Course of Preparing Elite Athletes for the Management of Upper Respiratory Infections during the COVID-19 Pandemic: A Narrative Review. Int. J. Environ. Res. Public Health.

[2] Timpka T. (2020). Sports Health during the SARS-CoV-2 Pandemic.

[3] Gabutti G., d’Anchera E., De Motoli F., Savio M., Stefanati A. (2021). The Epidemiological Characteristics of the COVID-19 Pandemic in Europe: Focus on Italy. Int. J. Environ. Res. Public Health.

[4] Mann R.H., Clift B.C., Boykoff J., Bekker S. (2020). Athletes as community; athletes in community: COVID-19, sporting mega-events and athlete health protection. Br. J. Sport. Med..

[5] Gallego V., Nishiura H., Sah R., Rodriguez-Morales A.J. (2020). The COVID-19 outbreak and implications for the Tokyo 2020 Summer Olympic Games. Travel Med. Infect. Dis..

[6] Gautret P., Al-Tawfiq J.A., Hoang V.T. (2020). COVID 19: Will the 2020 Hajj pilgrimage and Tokyo Olympic Games be cancelled?. Travel Med. Infect. Dis..

[7] Carmody S., Murray A., Borodina M., Gouttebarge V., Massey A. (2020). When can professional sport recommence safely during the COVID-19 pandemic? Risk assessment and factors to consider. Br. J. Sport. Med..

[8] Hull J.H., Loosemore M., Schwellnus M. (2020). Respiratory health in athletes: Facing the COVID-19 challenge. Lancet Respir. Med..

[9] Cao X. (2020). COVID-19: Immunopathology and its implications for therapy. Nat. Rev. Immunol..

[10] Montazersaheb S., Hosseiniyan Khatibi S.M., Hejazi M.S., Tarhriz V., Farjami A., Ghasemian Sorbeni F., Farahzadi R., Ghasemnejad T. (2022). COVID-19 infection: An overview on cytokine storm and related interventions. Virol. J..

[11] Li X., Geng M., Peng Y., Meng L., Lu S. (2020). Molecular immune pathogenesis and diagnosis of COVID-19. J. Pharm. Anlysis.

[12] Costela-Ruiz V.J., Illescas-Montes R., Puerta-Puerta J.M., Ruiz C., Melguizo-Rodríguez L. (2020). SARS-CoV-2 infection: The role of cytokines in COVID-19 disease. Cytokine Growth Factor Rev..

[13] Sellami M., Al-Muraikhy S., Al-Jaber H., Al-Amri H., Al-Mansoori L., Mazloum N.A., Donati F., Botre F., Elrayess M.A. (2021). Age and Sport Intensity-Dependent Changes in Cytokines and Telomere Length in Elite Athletes. Antioxidants.

[14] Suzuki K., Hekmatikar A.H.A., Jalalian S., Abbasi S., Ahmadi E., Kazemi A., Ruhee R.T., Khoramipour K. (2022). The Potential of Exerkines in Women’s COVID-19: A New Idea for a Better and More Accurate Understanding of the Mechanisms behind Physical Exercise. Int. J. Environ. Res. Public Health.

[15] Fischer J.C., Zanker K., van Griensven M., Schneider M., Kindgen-Milles D., Knoefel W.T., Lichtenberg A., Tamaskovics B., Djiepmo-Njanang F.J., Budach W. (2020). The role of passive immunization in the age of SARS-CoV-2: An update. Eur. J. Med. Res..

[16] Saverino D. (2020). Hyper-immune/convalescent plasma: An old option and a valid strategy for treatment of COVID-19?. Minerva Med..

[17] Sheridan C. (2020). Convalescent serum lines up as first-choice treatment for coronavirus. Nat. Biotechnol..

[18] Ye M., Fu D., Ren Y., Wang F., Wang D., Zhang F., Xia X., Lv T. (2020). Treatment with convalescent plasma for COVID-19 patients in Wuhan, China. J. Med. Virol..

[19] Zhang B., Liu S., Tan T., Huang W., Dong Y., Chen L., Chen Q., Zhang L., Zhong Q., Zhang X. (2020). Treatment with Convalescent Plasma for Critically Ill Patients with SARS-CoV-2 Infection. Chest.

[20] Shen C., Wang Z., Zhao F., Yang Y., Li J., Yuan J., Wang F., Li D., Yang M., Xing L. (2020). Treatment of 5 Critically Ill Patients With COVID-19 With Convalescent Plasma. JAMA.

[21] Duan K., Liu B., Li C., Zhang H. (2020). Effectiveness of convalescent plasma therapy in severe COVID-19 patients. Proc. Natl. Acad. Sci. USA.

[22] Rojas M., Rodríguez Y., Monsalve D.M., Acosta-Ampudia Y., Camacho B., Gallo J.E., Rojas-Villarraga A., Ramírez-Santana C., Díaz-Coronado J.C., Manrique R. (2020). Convalescent plasma in COVID-19: Possible mechanisms of action. Autoimmun. Rev..

[23] di Mauro G., Scavone C., Rafaniello C., Rossi F., Capuano A. (2020). SARS-CoV-2 infection: Response of human immune system and possible implications for the rapid test and treatment. Int. Immunopharmacol..

[24] Kim K.D., Hwang I., Ku K.B., Lee S., Kim S.J., Kim C. (2020). Progress and Challenges in the Development of COVID-19 Vaccines and Current Understanding of SARS-CoV-2-Specific Immune Responses. J. Microbiol. Biotechnol..

[25] Mendoza R., Silver M., Zuretti A.R., Christian M., Das B., Norin A.J., Borgen P., Libien J., Bluth M.H. (2021). Correlation of Automated Chemiluminescent Method with Enzyme-Linked Immunosorbent Assay (ELISA) Antibody Titers in Convalescent COVID-19 Plasma Samples: Development of Rapid, Cost-Effective Semi-Quantitative Diagnostic Methods. J. Blood Med..

[26] Zhou W., Xu X., Chang Z., Wang H., Zhong X., Tong X., Liu T., Li Y. (2021). The dynamic changes of serum IgM and IgG against SARS-CoV-2 in patients with COVID-19. J. Med. Virol..

[27] Horspool A.M., Kieffer T., Russ B.P., DeJong M.A., Wolf M.A., Karakiozis J.M., Hickey B.J., Fagone P., Tacker D.H., Bevere J.R. (2021). Interplay of Antibody and Cytokine Production Reveals CXCL13 as a Potential Novel Biomarker of Lethal SARS-CoV-2 Infection. MSphere.

[28] Olmos Calvo I., Kuten-Pella O., Kramer K., Madár Á., Takács S., Kardos D., Simon D., Erdö-Bonyár S., Berki T., De Luna A. (2021). Optimization of Lyophilized Hyperacute Serum (HAS) as a Regenerative Therapeutic in Osteoarthritis. Int. J. Mol. Sci..

[29] Petrone L., Petruccioli E., Vanini V., Cuzzi G., Fard S.N., Alonzi T., Castilletti C., Palmieri F., Gualano G., Vittozzi P. (2021). A whole blood test to measure SARS-CoV-2-specific response in COVID-19 patients. Clin. Microbiol. Infect. Off Publ. Eur. Soc. Clin. Microbiol. Infect. Dis..

[30] Bonny T.S., Patel E.U., Zhu X., Bloch E.M., Grabowski M.K., Abraham A.G., Littlefield K., Shrestha R., Benner S.E., Laeyendecker O. (2021). Cytokine and Chemokine Levels in Coronavirus Disease 2019 Convalescent Plasma. Open Forum Infect. Dis..

[31] Petruccioli E., Fard S.N., Navarra A., Petrone L., Vanini V., Cuzzi G., Gualano G., Pierelli L., Bertoletti A., Nicastri E. (2021). Exploratory analysis to identify the best antigen and the best immune biomarkers to study SARS-CoV-2 infection. J. Transl. Med..

[32] Tarantino U., Visconti V.V., Bonanni R., Gatti A., Marcozzi M., Calabrò D., Cariati I. (2022). Osteosarcopenia and Long-COVID: A dangerous combination. Ther. Adv. Musculoskelet. Dis..

[33] Huang C., Huang L., Wang Y., Li X., Ren L., Gu X., Kang L., Guo L., Liu M., Zhou X. (2021). 6-month consequences of COVID-19 in patients discharged from hospital: A cohort study. Lancet.

[34] Xie Y., Xu E., Bowe B., Al-Aly Z. (2022). Long-term cardiovascular outcomes of COVID-19. Nat Med..

